# Supplementary material for: Prenatal lead exposure is negatively associated with the gut microbiome in childhood
Source: Front Microbiol. 2023 Jun 22;14:1193919. doi: 10.3389/fmicb.2023.1193919 (PMC10325945; doi:10.3389/fmicb.2023.1193919)
Supplement: Supplementary file 1 [file Table_1.pdf]

Supplementary Table S1. The 13 variables included in the derivation of the socio-economic status (SES) variable used in this analysis.

Variable	Label
Bathrooms	Number of bathrooms with shower
Boiler	Do you have a boiler? 0=No, 1=Yes
Car	Number of cars
Children	Number of children
Comp	Do you have a personal computer? 0=No, 1=Yes
Floor	Type of flooring: 0=Ground or cement, 1=Other material
Lightbulbs	Number of lightbulbs: 1= 5 or less, 2= 6-10, 3= 11-15, 4=16-20, 5= 21 or more
Micro	Do you have a microwave? 0=No, 1=Yes
Player	Do you have a video or DVD player? 0=No, 1=Yes
Rooms	Number of rooms
Toast	Do you have a toaster? 0=No, 1=Yes
Vacuum	Do you have a vacuum cleaner? 0=No, 1=Yes
Washer	Do you have a washing machine? 0=No, 1=Yes
