# Supplementary material for: Prenatal lead exposure is negatively associated with the gut microbiome in childhood
Source: Front Microbiol. 2023 Jun 22;14:1193919. doi: 10.3389/fmicb.2023.1193919 (PMC10325945; doi:10.3389/fmicb.2023.1193919)
Supplement: Supplementary file 2 [file Table_2.pdf]

Supplementary Table S2: Top 20 microbial metabolic pathways of highly weighted taxa by trimester of prenatal Pb exposure.

Second trimester Pb	Third trimester Pb
PWY-7219: adenosine ribonucleotides de novo biosynthesis	PWY-7219: adenosine ribonucleotides de novo biosynthesis
PWY-7221: guanosine ribonucleotides de novo biosynthesis	COA-PWY-1: coenzyme A biosynthesis II (mammalian)
COA-PWY-1: coenzyme A biosynthesis II (mammalian)	PWY-7221: guanosine ribonucleotides de novo biosynthesis
HISTSYN-PWY: L-histidine biosynthesis	PWY-6122: 5-aminoimidazole ribonucleotide biosynthesis II
PWY-5686: UMP biosynthesis	PWY-6277: superpathway of 5-aminoimidazole ribonucleotide biosynthesis
PWY-6122: 5-aminoimidazole ribonucleotide biosynthesis II	PWY-6121: 5-aminoimidazole ribonucleotide biosynthesis I
PWY-6277: superpathway of 5-aminoimidazole ribonucleotide biosynthesis	PWY-6151: S-adenosyl-L-methionine cycle I
PWY-6897: thiamin salvage II	PWY-6700: queuosine biosynthesis
PWY-7111: pyruvate fermentation to isobutanol (engineered)	PANTO-PWY: phosphopantothenate biosynthesis I
PWY-7357: thiamin formation from pyrithiamine and oxythiamine (yeast)	PEPTIDOGLYCANSYN-PWY: peptidoglycan biosynthesis I (meso-diaminopimelate containing)
VALSYN-PWY: L-valine biosynthesis	PWY-3841: folate transformations II
BRANCHED-CHAIN-AA-SYN-PWY: superpathway of branched amino acid biosynthesis	PWY-5100: pyruvate fermentation to acetate and lactate II
COA-PWY: coenzyme A biosynthesis I	PWY-6386: UDP-N-acetylmuramoyl-pentapeptide biosynthesis II (lysine-containing)
DTDPRHAMSYN-PWY: dTDP-L-rhamnose biosynthesis I	PWY-6387: UDP-N-acetylmuramoyl-pentapeptide biosynthesis I (meso-diaminopimelate containing)
HSERMETANA-PWY: L-methionine biosynthesis III	PWY-7111: pyruvate fermentation to isobutanol (engineered)
ILEUSYN-PWY: L-isoleucine biosynthesis I (from threonine)	VALSYN-PWY: L-valine biosynthesis
LACTOSECAT-PWY: lactose and galactose degradation I	COA-PWY: coenzyme A biosynthesis I
METHANOGENESIS-PWY: methanogenesis from H2 and CO2	DTDPRHAMSYN-PWY: dTDP-L-rhamnose biosynthesis I
PWY-2941: L-lysine biosynthesis II	LACTOSECAT-PWY: lactose and galactose degradation I
PWY-2942: L-lysine biosynthesis III	NONMEVIPP-PWY: methylerythritol phosphate pathway I
