# Supplementary material for: Prenatal lead exposure is negatively associated with the gut microbiome in childhood
Source: Front Microbiol. 2023 Jun 22;14:1193919. doi: 10.3389/fmicb.2023.1193919 (PMC10325945; doi:10.3389/fmicb.2023.1193919)
Supplement: Supplementary file 3 [file Image_1.pdf]

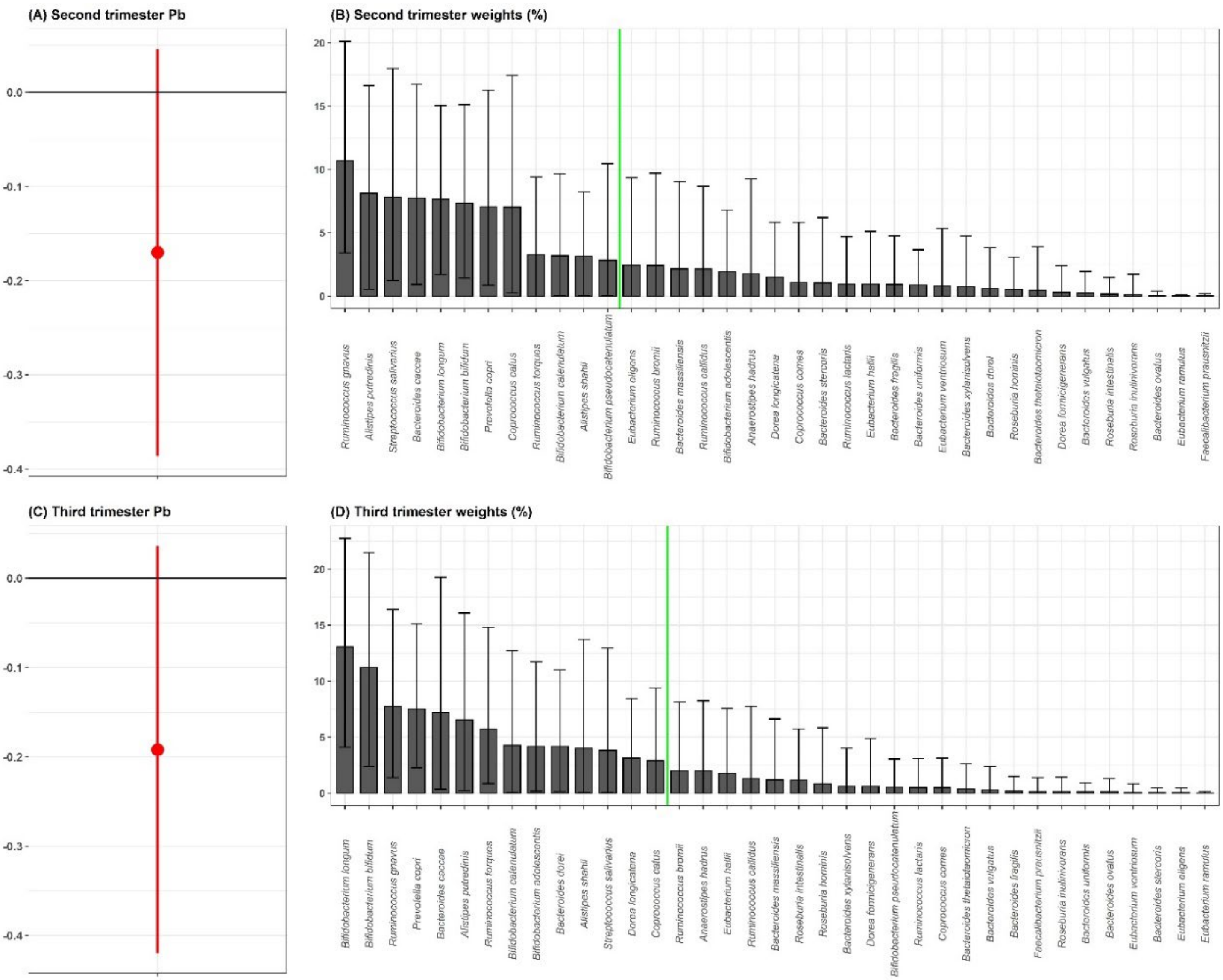

Supplementary Figure S1. Sensitivity analysis WQS_{RSRH} estimates for the association of the gut microbiome mixture with prenatal Pb exposure in the a) second and c) third trimester of pregnancy, including only the taxa that are present in at least 25% of participants from each analytical batch. Average percent weight for each taxa within the WQS index are shown for the b) second and d) third trimester Pb exposure. The green line indicates the importance threshold for weights above random chance.
